# Identifying the training needs of Australian primary health professionals to support early childhood obesity prevention: a mixed methods study

**DOI:** 10.1017/S146342362610108X

**Published:** 2026-03-27

**Authors:** Eve T. House, Erin Kerr, Sarah Taki, Elizabeth Denney-Wilson, Louise A. Baur, Heilok Cheng, Chris Rossiter, Sharlene Vlahos, Li Ming Wen

**Affiliations:** 1 The University of Sydney School of Public Health, Faculty of Medicine and Health, The University of Sydney, Sydney, NSW, Australia; 2 NHMRC Centre of Research Excellence in Translating Early Prevention of Obesity in Childhood (EPOCH-Translate CRE), Sydney, NSW, Australia; 3 Health Promotion Unit, Population Health Research & Evaluation Hub, Sydney Local Health District, Sydney, NSW, Australia; 4 Sydney Institute for Women, Children and Their Families, Sydney Local Health District, NSW Health, Sydney, NSW, Australia; 5 Susan Wakil School of Nursing and Midwifery, Faculty of Medicine and Health, The University of Sydney, Sydney, NSW, Australia; 6 Sydney Medical School, The University of Sydney, Sydney, NSW, Australia; 7 Karitane, Villawood, NSW, Australia

**Keywords:** Child, continuing professional development, infant, obesity, Primary health care

## Abstract

**Objectives:**

Practice guidelines for Australian primary health professionals (PHPs) highlight their crucial role in preventive care. However, PHPs report a lack of knowledge and skills regarding early childhood obesity prevention. This study aimed to identify the training needs of Australian PHPs – including child and family health nurses (CFHNs), general practitioners, general practice nurses and other community-based health professionals – to support early childhood health promotion and obesity prevention.

**Methods:**

From August 2022 to July 2023, PHPs were recruited to participate in an online survey and semi-structured interviews. Quantitative data was analysed descriptively and qualitative data analysed using reflexive thematic analysis.

**Results:**

227 PHPs returned a survey (46% CFHNs) and 28 were interviewed (13 CFHNs). Almost a quarter (23%) of participants had not received any continuing education regarding early childhood health behaviours and obesity prevention, with general practice professionals less likely to have participated in such education. PHPs identified a need to develop skills in growth assessment and working with children at risk of obesity. Digital and visual parent-facing resources were required to support PHPs’ discussions of child health behaviours. Important components of education were case studies, self-paced learning, and live interactive discussions (37–46% of PHPs rated as highly important). PHPs sought interactive education activities from reputable service providers and reported time and cost were barriers to education.

**Conclusions:**

Australian PHPs require access to evidence-based education and resources to support early childhood health promotion and obesity prevention. Professional education providers should prioritize interactive and flexible modes of delivery.

## Introduction

Establishing healthy behaviours in early life sets the stage for healthy growth trajectories and prevention of later disease (Mihrshahi and Baur, 2018). The Australian National Obesity Strategy 2022–2032 recognizes the importance of obesity prevention in early life and highlights support for parents to establish healthy eating, sleep and activity behaviours for their children as a policy priority (Commonwealth of Australia, 2022). The policy recommends a multifaceted approach to obesity prevention in the first 2,000 days, from conception to age five years, with involvement of health promotion across multiple early childhood settings, including all levels of healthcare (Commonwealth of Australia, 2022).

Within Australian primary healthcare, health promotion is embedded in practice guidelines for primary health professionals (PHPs). Child and Family Health Nurses (CFHNs) work across various settings in Australia to deliver primary healthcare services to children in the first five years of life. Health promotion is a core component of the CFHN role – particularly, understanding infant and child growth and development, and addressing parents’ concerns related to feeding, nutrition, sleep and settling (NSW Child and Family Health Nursing Clinical Nurse Consultant Network and Child and Family Health Nurses Association (NSW), 2022, Grant *et al.*, 2017). Similarly, preventive care guidelines for general practitioners (GPs) encourage GPs to promote healthy weight, nutrition and exercise during pregnancy, positive infant feeding practices, healthy eating, physical activity, and quality sleep throughout early childhood (Royal Australian College of General Practitioners, 2024). Likewise, practice standards for general practice nurses highlight their role in health promotion and preventive care (Australian Nursing and Midwifery Federation, 2014).

Since preventive care has been identified as a core component of primary healthcare activities, PHPs might be expected to have received adequate training to support early childhood health promotion. The tertiary curriculum for CFHNs typically includes feeding, nutrition and growth (Maternal Child and Family Health Nurses Australia, 2024) – however, studies have found that CFHNs report requiring further information to support obesity prevention in early childhood (Cheng *et al.*, 2020, Laws *et al.*, 2015). GPs receive limited training related to nutrition: medical educators report that nutritional training in the general practice curriculum is limited (Ball *et al.*, 2010), with a 2019 review identifying a lack of nutritional training in medical education more broadly (Crowley *et al.*, 2019). Notably, a 2022 systematic review explored barriers and facilitators to early childhood obesity prevention in primary healthcare settings (Ray *et al.*, 2022). This review identified that PHPs perceived they lacked knowledge and skills to support health promotion activities including breastfeeding support; use of body mass index (BMI) thresholds to guide growth assessment and sensitively discuss children’s weight; and knowledge of risk factors for excess weight gain and the guidelines for health behaviours (such as nutrition, physical activity and sedentary behaviour) (Ray *et al.*, 2022).

Continuing professional development (CPD) can develop the knowledge, skills and practices of health professionals beyond their formal undergraduate and postgraduate studies (Cervero and Gaines, 2015, Gijbels *et al.*, 2010). Adult education programmes must consider the needs of the adult learner to be effective (Knowles *et al.*, 2015). Thus, this study sought to identify the training needs of Australian PHPs to support health promotion and obesity prevention for children aged 0–5 years.

## Materials and methods

This mixed methods study was informed by a critical realist perspective, a view that there exists an independent reality, but experiences of reality are shaped by contextual factors, such as language and culture (Braun and Clarke, 2022, Koopmans and Schiller, 2022). Thus, in this study, we used quantitative data collected in an online survey to understand the nature of PHPs’ previous training and future preferences, and qualitative data collected in semi-structured interviews were used to explore the underlying factors that may contribute to PHPs’ engagement or lack of engagement with training programmes and resources. The methods have been described in detail elsewhere (House *et al.,* 2025) and are described in brief below.

### Participants

We sought to recruit 200 PHPs to participate in the survey and 20 to participate in an interview. Eligible participants were PHPs practising in Australia working with pregnant women and children 0–5 years. They included GPs, CFHNs, general practice nurses (GPNs), and other health professionals working in community settings.

### Recruitment

Recruitment occurred from August 2022 to July 2023, with recruitment material distributed via email to Australian professional organizations (including Primary Health Networks and professional and community health organizations for GPs, primary health nurses, and CFHNs) and on social media. Recruitment material directed participants to the Participant Information Sheet and online consent form. Prior to completing the consent form, prospective participants were asked to complete one screening question ‘Are you a primary health care professional who supports families with children aged 0–5 years?’ to assess eligibility for the survey. At the end of the survey, participants could express interest in interview participation, and recruitment material targeting GPs provided the option to participate in an interview with or without survey participation. Purposive sampling was used to attempt to contact interested interviewees from different professional groups (i.e. GPs, GPNs, CFHNs, other PHPs), with a range of levels of experience, and working across different states, and in a mix of metropolitan, regional, and remote areas with varying levels of socioeconomic disadvantage.

### Survey

The 20-item survey was adapted from previous research (Laws *et al.*, 2015) and distributed online using REDCap, a secure research data management platform (Harris *et al.*, 2019, Harris *et al.*, 2009). The twelve questions included in this analysis addressed demographics, PHPs’ access to relevant guidelines and resources, engagement with CPD related to early childhood health behaviours and growth, and preferred topics and features of future CPD programmes (Appendix 1).

### Interview

Semi-structured interviews were conducted, via telephone or video conference. Interview questions focussed on PHPs’ needs and preferences for CPD, and resources related to early childhood health promotion (Appendix 1). One researcher (EH – a research dietitian and PhD candidate without clinical experience in primary healthcare), with no previous relationship with participants, conducted the interviews after obtaining verbal or electronic informed consent. Interviews were recorded using Zoom and transcribed using Microsoft Word transcription functions, with transcripts cross-checked for accuracy by EH.

### Analysis

REDCap survey responses were reviewed against a data cleaning protocol and excluded if they met criteria indicative of a high likelihood of illegitimacy (Appendix 2). Descriptive analysis of included surveys was conducted using IBM SPSS Statistics V29.0 (Chicago, IL). The index of relative socioeconomic disadvantage (IRSD) for the area where PHPs worked was derived from their workplace postcode, areas were categorized as low (IRSD 1–3), middle (IRSD 4–7) or high (IRSD 8–10) socioeconomic status (SES) (Australian Bureau of Statistics, 2021). Variables relating to the importance of features of CPD programmes were recoded into three categories – low (‘not very’ important), medium (‘somewhat’ or ‘moderately’ important), and high (‘very’ important) importance. Open-text responses, regarding PHPs’ use of guidelines and the resources they required, were categorized, with frequencies and percentages reported. PHPs’ access to resources, use of guidelines, previous CPD and interest in future CPD topics were compared between CFHNs and general practice PHPs (i.e. GPNs and GPs) using Chi-squared or Fisher’s exact tests – PHPs in other roles were excluded from sub-group analysis.

Interviews were transcribed verbatim, and transcripts were entered into Lumivero (2023) *NVivo* (Version 14). Reflexive thematic analysis of interviews was conducted, following a six-phase framework (Braun and Clarke, 2022). Themes were mapped to the drivers of behaviour described in the COM-B model of behaviour change i.e. capability, opportunity and motivation (Michie *et al.*, 2014). The COM-B model offers a useful framework for identifying key opportunities for intervention development to facilitate PHP behaviour change.

### Ethics

This study was approved by the Sydney Local Health District Human Research Ethics Committee (RPAH Zone) (Protocol number X22–0022). Prior to completing the survey, all participants provided written, informed consent electronically in REDCap. Interview participants provided either written or verbal informed consent. Written consent was provided electronically in REDCap, verbal consent was obtained and recorded by the researcher prior to commencing the interview.

## Results

### Survey results

#### Survey participants

There were 273 survey responses recorded; 227 PHPs’ survey responses were included in the analysis, and 46 responses were excluded during data cleaning. Of 187 PHPs who reported their profession, the largest group were CFHNs (*n* = 104, 56% of those providing demographic information), most were female (95% of those providing demographic information) and practising in New South Wales (59% of those providing demographic information) (Appendix 3 – Supplementary Table 1a).

#### Access to relevant resources and use of professional guidelines

Compared to CFHNs, general practice PHPs were less likely to use published guidelines to inform their advice and practice regarding infant feeding, growth monitoring and early childhood health promotion (Table 1). Twenty-nine PHPs reported the type of guidelines they used, with national (*n* = 16) and state guidelines (*n* = 15) used most frequently, and international (*n* = 4) and local (*n* = 5) guidelines used less frequently.

**Table 1.** The use of published guidelines to inform practice and advice among primary health professionals and its comparisons between Child and Family Health Nurses and professionals in general practice

Guideline Topic	PHPs using published guidelines to inform practice and advice				
	All participants (*n* = 227)	CFHNs (*n* = 104)	PHPs in general practice (*n* = 50)	Difference between settings	
	*N* (%)	*N* (%)	*N* (%)	*p*-value	Phi coefficient
Infant feeding	149 (66%)	96 (92%)	24 (48%)**	<0.001	0.500
Growth monitoring in infants (0–2 years)	118 (52%)	80 (77%)	16 (32%)**	<0.001	0.434
Growth monitoring in children (2–5 years)	97 (43%)	68 (65%)	11 (22%)**	<0.001	0.406
Healthy eating in young children (0–5 years)	130 (57%)	81 (78%)	21 (42%)**	<0.001	0.355
Physical activity in young children (0–5 years)	76 (33%)	53 (51%)	6 (12%)**	<0.001	0.375
Sedentary behaviour in young children (0–5 years)	53 (23%)	37 (36%)	4 (8%)**	<0.001	0.292
None of the above	29 (13%)	5 (5%)	20 (40%)**	<0.001	0.447

**p < 0.001 when compared to child and family health nurses using Chi-squared or Fisher’s exact test.

CFHN – Child and Family Health Nurse, PHP – primary health professional.

Most PHPs could access resources to measure and plot growth. Compared to CFHNs, general practice PHPs were less likely to have access to a recumbent length board and education materials regarding early childhood health behaviours, including culturally adapted resources (Table 2a). PHPs reported a need for resources to support growth monitoring and assessment in early childhood. PHPs also required more resources for families from culturally and linguistically diverse and First Nations backgrounds (Table 2b).

**Table 2.** (a) Access to resources among primary health professionals and its comparisons between Child and Family Health Nurses and professionals in general practice (b) Types of resources required when working with infants and young children as reported by primary health professionals

	PHPs with access to resources by setting				
	All participants (*n* = 227)	CFHNs (*n* = 104)	PHPs in general practice (*n* = 50)	Difference between settings	
a) Resources	*N* (%)	*N* (%)	*N* (%)	*p*-value	Phi coefficient
Growth charts for infants	210 (93%)	103 (99%)	48 (96%)	0.246	0.103
BMI percentile chart for children	189 (83%)	92 (88%)	44 (88%)	0.933	0.007
Education materials about infant feeding	176 (78%)	99 (95%)	24 (48%)**	<0.001	0.551
Education materials about healthy eating for toddlers and preschoolers	158 (70%)	92 (88%)	20 (40%)**	<0.001	0.510
Education materials about promoting active play	128 (56%)	83 (80%)	9(18%)**	<0.001	0.590
Education materials about limiting sedentary behaviour	104 (46%)	67 (64%)	9 (18%)**	<0.001	0.435
Education materials about sleep and settling	146 (64%)	95 (91%)	12 (24%)**	<0.001	0.685
Scales to measure infant weight	205 (90%)	101 (97%)	49 (98%)	1.000	0.026
Scales to measure child weight	201 (89%)	98 (94%)	48 (96%)	1.000	0.037
Recumbent length board	197 (87%)	102 (98%)	45 (90%)*	0.037	0.182
Education materials for culturally and linguistically diverse families	104 (46%)	63 (61%)	9 (18%)**	<0.001	0.400

b) Resources required when working with infants (0–2 years) (*n* = 82 responses)			*N* (%)		
**Format**					
Digital			10 (12%)		
Handouts			16 (20%)		
Visual			12 (15%)		
**Topic Area**					
Food preparation			3 (4%)		
Food allergies and allergens			2 (2%)		
Bottle-feeding and cup transition			9 (11%)		
Breastfeeding			3 (4%)		
Growth monitoring and assessment			15 (18%)		
Physical activity			4 (5%)		
Healthy eating			7 (9%)		
Sedentary behaviour			7 (9%)		
Fussy eating behaviours			6 (7%)		
Nutritional requirements			9 (11%)		
Special diets (e.g. vegetarian)			2 (2%)		
Introducing solids			6 (7%)		
Responsive feeding			3 (4%)		
**Other resourcing**					
Resources for culturally and linguistically diverse families			7 (9%)		
Resources for First Nations families			3 (4%)		
Resources in collated/easy-to-access location			3 (4%)		
Health professional training opportunities			3 (4%)		
Additional time			3 (4%)		
Multidisciplinary referral options			2 (2%)		
Updates to guidelines			2 (2%)		
None/not sure			7 (9%)		

Resources required when working with young children (2–5 years) (*n* = 78 responses)			*N* (%)		
**Format**					
Digital			12 (15%)		
Handouts			15 (19%)		
Visual			12 (15%)		
**Topic Area**					
Food preparation			1 (1%)		
Food allergies and allergens			1 (1%)		
Bottle-feeding and cup transition			3 (4%)		
Growth monitoring and assessment			16 (21%)		
Physical activity			6 (8%)		
Healthy eating			11 (14%)		
Sedentary behaviour			7 (9%)		
Fussy eating behaviours			8 (10%)		
Nutritional requirements			7 (9%)		
Special diets (e.g. vegetarian)			3 (4%)		
Introducing solids			1 (1%)		
Responsive feeding			2 (3%)		
Label reading			1 (1%)		
**Other resourcing**					
Resources for culturally and linguistically diverse families			6 (8%)		
Resources for First Nations families			2 (3%)		
Resources for vulnerable families			1 (1%)		
Resources in collated/easy-to-access location			3 (4%)		
Health professional training opportunities			3 (4%)		
Additional time			4 (5%)		
Multidisciplinary referral options			5 (6%)		
Updates to guidelines			1 (1%)		
Government support for prevention			1 (1%)		
None/not sure			8 (10%)		

*p < 0.05 when compared to child and family health nurses using Chi-squared or Fisher’s exact test.

**p < 0.001 when compared to child and family health nurses using Chi-squared or Fisher’s exact test.

BMI – body mass index, CFHN – Child and Family Health Nurse, PHP – primary health professional.

#### Previous professional education

Three-quarters of participants reported previous participation in CPD regarding early childhood nutrition and related health promotion topics in the past two years. More CFHNs had participated in CPD regarding breastfeeding and starting solids compared to general practice PHPs. Few PHPs had attended CPD about limiting sedentary behaviour, obesity management, communication with parents about the risk of overweight and obesity, active play, and behaviour change techniques (Table 3).

**Table 3.** Received training related to early childhood obesity prevention in the past two years as reported by primary health professionals, and its comparisons between Child and Family Health Nurses and professionals in general practice

	PHPs who have received training in the past 2 years (n(%))				
	All respondents (*n* = 227)	CFHNs (*n* = 104)	PHPs in general practice (*n* = 50)	Difference between settings	
Topic	*N* (%)	*N* (%)	*N* (%)	*p*-value	Phi co-efficient
Breastfeeding	112 (49%)	77 (74%)	17 (34%)**	<0.001	0.384
Best practice formula feeding	38 (17%)	22 (21%)	5 (10%)	0.088	0.137
Introduction of solids to infants	64 (28%)	41 (39%)	11 (22%)*	0.032	0.173
Healthy infant feeding practices	45 (20%)	23 (22%)	9 (18%)	0.556	0.047
Healthy eating for young children	51 (22%)	27 (26%)	10 (20%)	0.417	0.065
Active play for young children	31 (14%)	21 (20%)	7 (14%)	0.351	0.075
Limiting sedentary behaviour in young children	22 (10%)	12 (12%)	7 (14%)	0.664	0.035
Obesity prevention in children	42 (19%)	23 (22%)	7 (14%)	0.234	0.096
Obesity management in children	22 (10%)	11 (11%)	5 (10%)	0.913	0.009
Behaviour change techniques	33 (15%)	17 (16%)	10 (20%)	0.577	0.045
Monitoring infant growth and identifying those at risk of overweight	50 (22%)	32 (31%)	8 (16%)	0.050	0.158
Communication with parents about the risks for later overweight and obesity	30 (13%)	19 (18%)	6 (12%)	0.323	0.080
None of the above	53 (23%)	19 (18%)	23 (46%)**	<0.001	0.292

*p < 0.05 when compared to child and family health nurses using Chi-squared or Fisher’s exact test.

**p < 0.001 when compared to child and family health nurses using Chi-squared or Fisher’s exact test.

CFHN – Child and Family Health Nurse, PHP – primary health professional.

#### Topics of interest for future CPD

Most PHPs reported they would be interested in online CPD regarding infant and child nutrition. Topics of most interest included fussy eating, allergies and food intolerances, providing culturally appropriate nutrition advice to families, nutrients of concern in pregnancy, childhood and special diets, and preventing childhood obesity. General practice PHPs were less likely than CFHNs to report interest in CPD regarding providing culturally appropriate nutrition support to families, and motivational interviewing (Table 4).

**Table 4.** Topics of interest for continuing professional development related to early childhood obesity prevention and health promotion among primary health professionals and its comparisons between Child and Family Health Nurses and professionals in general practice

	PHPs with interest in training on the topic (n(%))				
	All participants (*n* = 227)	CFHNs (*n* = 104)	PHPs in general practice (*n* = 50)	Difference between settings	
Topic	*N* (%)	*N* (%)	*N* (%)	*p*-value	Phi coefficient
Fussy eating	136 (60%)	82 (79%)	34 (68%)	0.144	0.118
Introduction to solids	65 (29%)	32 (31%)	22 (44%)	0.107	0.130
Breastfeeding	60 (26%)	28 (27%)	21 (42%)	0.060	0.152
Best practice formula feeding	95 (42%)	49 (47%)	30 (60%)	0.134	0.121
Prenatal and preconception nutrition	60 (26%)	28 (27%)	21 (42%)	0.060	0.152
Nutrients of concern for pregnancy/children/special diets	112 (49%)	67 (64%)	26 (52%)	0.140	0.119
Allergies and intolerances	126 (56%)	74 (71%)	35 (70%)	0.883	0.012
Baby-led weaning	86 (38%)	52 (50%)	20 (40%)	0.244	0.094
Preventing childhood obesity	109 (48%)	64 (62%)	28 (56%)	0.512	0.053
Talking to parent/carers about their child’s weight	95 (42%)	58 (56%)	23 (46%)	0.256	0.092
Providing culturally appropriate nutrition advice and support to families	114 (50%)	75 (72%)	22 (44%)**	<0.001	0.273
Communicating nutrition information to parents/carers	75 (33%)	43 (41%)	20 (40%)	0.874	0.013
Motivational interviewing	82 (36%)	53 (51%)	17 (34%)*	0.048	0.160
Measuring infants’ height and weight and plotting on a growth chart	27 (12%)	13 (13%)	7 (14%)	0.795	0.021
Identifying infants who are at risk of overweight or obesity	91 (40%)	52 (50%)	28 (56%)	0.485	0.056
Identifying young children who are at risk of overweight or obesity	89 (39%)	47 (45%)	31 (62%)	0.051	0.157

*p < 0.05 when compared to child and family health nurses using Chi-squared or Fisher’s exact test.

**p < 0.001 when compared to child and family health nurses using Chi-squared or Fisher’s exact test.

CFHN – Child and Family Health Nurse, PHP – primary health professional.

#### Preferred features of CPD programmes

When asked to rate the importance of features of CPD programmes, the largest proportion of participants rated the use of case studies (46%), self-paced learning (39%), and live interactive discussion (37%) as highly important. Quizzes were most often rated as low importance (15% rated as low importance) (Table 5).

**Table 5.** Health professional ratings of the importance of different features of professional development programmes

	PHPs rating of importance of CPD features		
	All participants (*n* = 227)	CFHNs (*n* = 104)	PHPs in general practice (*n* = 50)
	*N* (%)^a^	*N* (%)^a^	*N* (%)^a^
Case studies			
Low importance	7 (4%)	3 (3%)	4 (8%)
Medium importance	94 (50%)	46 (44%)	30 (60%)
High importance	87 (46%)	55 (53%)	16 (32%)
Live interactive discussion			
Low importance	8 (4%)	5 (5%)	2 (4%)
Medium importance	109 (59%)	55 (53%)	31 (63%)
High importance	68 (37%)	43 (42%)	16 (33%)
Quizzes			
Low importance	27 (15%)	13 (13%)	8 (16%)
Medium importance	135 (73%)	78 (76%)	35 (71%)
High importance	23 (12%)	11 (11%)	6 (12%)
Videos			
Low importance	3 (2%)	0 (0%)	3 (6%)
Medium importance	130 (70%)	73 (70%)	33 (67%)
High importance	54 (29%)	31 (30%)	13 (27%)
Opportunities to practise and enhance skills			
Low importance	6 (3%)	5 (5%)	1 (2%)
Medium importance	113 (61%)	54 (53%)	37 (74%)
High importance	67 (36%)	43 (42%)	12 (24%)
Self-paced learning			
Low importance	1 (1%)	1 (1%)	0 (0%)
Medium importance	114 (61%)	62 (60%)	33 (66%)
High importance	73 (39%)	41 (39%)	17 (34%)

CPD – continuing professional development, CFHN – Child and Family Health Nurse, PHP – primary health professional.

a. Percentage refers to the proportion of participants who answered each question, this differs by feature and ranged from 185–188 professionals (including 102–104 CFHNs and 49–50 PHPs in general practice).

### Interview results

Twenty-eight PHPs were interviewed, described in Appendix 3 – Supplementary Table 1b. Five themes were mapped to the drivers of behaviour described in the COM-B model (Michie *et al.*, 2014) – capability, opportunity and motivation.

#### Psychological capability: Equip professionals with the knowledge and skills to confidently promote health

Some PHPs, particularly in general practice, reported they had received minimal education regarding early childhood health behaviours:

*“It’s [childhood nutrition] not part of our regular medical school […] it’s not focused on much when you’re a registrar. It is not focused on at all after you finish, because hardly anybody talks about it in any of the CPD meetings at all.”* (P20, GP)

*“It’s [childhood nutrition] probably something that nurses in general practice don’t have a lot of confidence around unless they’ve come from like a paediatric background, and/or they go on what it was like raising their own children”* (P21, GPN)

Other PHPs reported having attended CPD activities regarding early childhood nutrition, either within their workplace or during their professional training:

*“Our community dietitians do a talk with us [...] they would usually focus on, like a certain topic, they don’t always cover breastfeeding. It would be more actual eating and nutrition. They also send out a monthly newsletter.” (P22, CFHN)*

*“The only extra training I got before fellowship was, like many GPs, I did the Diploma of Child Health”* (P26, GP)

All PHPs highlighted topics where they felt that additional CPD would be helpful, including formula feeding, introduction of solids, food allergies, and fussy eating:

*“I have a lot of clients that are talking to me and asking me about different formulas, so I don’t have a lot of understanding or information on the difference in the formulas”* (P5, CFHN)

*“Baby-led weaning, which is like so common now […] I can’t really find any clinical framework for it, and I feel kind of almost like I’m missing a big part when I do that four-month solid talk ‘cause I still have this handout that just goes puree to solids.”* (P6, GP)

*“People are still very concerned around allergies, and I know a lot of that’s changed now about giving allergenic foods. Before it was like don’t give anything till after 12 months, now it’s like give them everything pretty much [...] For myself, […] I’m not always all over it.”* (P2, CFHN)

PHPs also highlighted a desire for CPD to support professional skills, including growth assessment and working sensitively with families of children with higher weight:

*“I know a lot of our staff would struggle with, I guess, what’s normal growth? […] People do like BMIs and things, and we don’t really have a lot of education around that.”* (P18, CFHN)

*“When there is a large BMI, or, you know, like, when children come in as overweight or obese [sic], I think that’s the trickiest, and just framing that in a positive way and not, you know, shaming or blaming”* (P4, CFHN)

Other PHPs required further information to assist them with identifying referral pathways for infants and children with nutrition and growth-related concerns:

*“For me the education is not so much, what is the right thing to do, because I probably roughly know what the right thing to do is, but it’s how to point people […] how to easily advise people where to find the reliable information on what is the right thing to do.”* (P3, GP)

PHPs recalled receiving training about culturally responsive care – however, this was non-specific to nutrition and related health behaviours, and was often focussed on supporting First Nations clients, then adapted for use with families from culturally and linguistically diverse backgrounds:

*“We do a lot of work on cultural safety and just general cultural awareness, particularly with Aboriginal patients and, but you know, also just the more generally culturally and linguistically diverse populations that we’ve got. We don’t do anything specific to nutrition.”* (P26, GP)

Many professionals highlighted a lack of CPD regarding providing culturally responsive health promotion advice to families:

*Interviewer: “Have you come across any professional development that you found helpful in adapting some of these health-promoting messages for different cultures?”*

*Professional: “No, no that’s just a flat out, no. I haven’t had anything”* (P21, GPN)

More experienced clinicians expressed a desire for CPD that provided updates on the latest evidence regarding early childhood feeding, nutrition and related topics, as opposed to training focussed on established guidelines and clinical skills:

*“I’m probably working with quite an experienced cohort, so I feel like […] we probably are on board with most of those things of what children need. But I guess it’s just keeping that information up to date around, you know, what resources are out there, and particularly […] when they’ve swapped things like allergies, you know, ‘no, you can’t have this’, then ‘you can have this’”* (P10, CFHN)

#### Psychological capability: parent resources to support health promotion discussions

PHPs identified a need for parent-facing resources to support health promotion discussions. Many of the topics where they identified gaps in resources aligned with those where they sought additional CPD, including formula feeding and the introduction of solids:

*“like finger food handouts, and, like, pictures that show side-by-side different textures for like everyday meals so that you can, you know, show parents.”* (P24, CFHN)

Resources were also needed for fussy eating, the nutritional content of food, children’s requirements, food preparation, and supporting discussions with families after identification of higher weight:

*“A lot of the time, just telling you, look your baby’s too big or whatever, it’s useless to identify it, it’s useless without having a follow-up sort of support. Yeah, so I think these days, it’d all have to be web-based. But for general practice to use in the consult, it’d have to be quick.”* (P3, GP)

*“Years ago, there was like simple recipe books that came out that just used simple ingredients that you may have at your home [...] We were able to kind of hand them out that and go ‘look, you can make this, this, and this if you’ve got this in your cupboard’”* (P10, CFHN)

#### Psychological capability: design resources for real parents

PHPs provided insights into the resource needs of their family clientele. Professionals reported sharing health promotion information with parents in various formats, including verbally, in handouts, posters and digital resources:

*“I have pamphlets in the room that I give out. I also often refer to the online resource, the Raising Children Network, because that one covers pregnancy, children, all the way up to adults.”* (P7, GP)

Many PHPs perceived parents to prefer digital resources to traditional handouts:

*“Having a good web online resource is good. Yeah, links to things are really good, people like to look on their phone I guess, or online rather than actually read something.’* (P22, CFHN)

Professionals highlighted a need for simple resources suitable for parents with varying literacy levels, and emphasized the need for more visual resources with practical information, or tactile, tangible resources that supported skill development:

*“A lot of the materials we have that are produced by our nutrition unit are fabulous. But they’re not glossy picture brochures, they’re wordy.”* (P19, CFHN)

*“A lot of our clients are quite visual, so we will find it easier to bring, we call it, our nutrition services provided us with a ‘feeding pack’ […] a sippy cup, and a spoon in it and some information from the dietitian service […] Some of them we’ll actually show them how to cut up a banana and give a baby you know, just using this example, finger foods.”* (P10, CFHN)

Some PHPs reported sufficient access to translated or culturally adapted resources to support families from culturally and linguistically diverse backgrounds, others highlighted this as an important gap in their resources:

*“Raising Children’s has got a couple of resources that have been translated. So, we use them a little bit. We’ve actually, to do with our Korean population, we’ve made a couple of our own where we’ve worked with a couple of different organisations to put together pictorial storyboards of things.” (P24, CFHN)*

*Interviewer: “Do you find that there are resources available to support those [culturally and linguistically diverse] families in regards to nutrition?”*

*Professional: “Ah, not that seem to tick the ‘culturally aware’ box”* (P26, GP)

#### Physical opportunity: practical CPD solutions for busy clinicians

When asked about the aspects of CPD, regardless of topic, that would influence their decision to attend, PHPs described ways that CPD could be delivered to meet their needs amidst a busy clinical schedule. Limited time impacted PHPs’ ability to attend CPD, they often expressed a preference for short sessions that could fit within their work schedule:

*“Something that is short, timewise. Like not an all-day activity, I guess, like one or two hours”* (P22, CFHN)

Particularly, general practice PHPs reported attending CPD outside of work hours to avoid lost revenue:

*“It’s always in the evening, so daytime is packed, so you really can’t even think about doing anything else so that’s not possible at all.”* (P20, GP)

Many PHPs sought out flexible education programmes: some expressed that self-paced education (e.g. with recorded content or online modules) better suited their needs. Several emphasized that access to education recordings was instrumental in their decision to register for CPD activities:

*“There’s a lot of online ones [training], but the ones that are appealing are the ones that you can rewatch”* (P22, CFHN)

Many clinicians expressed that online training was more accessible and helped them to fit CPD into their schedule. PHPs also stated that cost would impact their decision to attend CPD, with many reporting that out-of-pocket costs would limit their capacity to attend:

*“It’s been really good having the availability of online, because we’re in a rural area, having to travel, you know, can be quite costly and quite time-consuming. So, a lot of the online education has been fantastic.”* (P4, CFHN)

*“From a practice nurse perspective. No cost or low cost. Because a lot of our courses cost upwards of $300 and we just don’t do them, because we just don’t get paid enough to pay that money.”* (P25, GPN)

PHPs also reported that they would prioritize CPD where they could see direct relevance to their practice:

*“…relevance to the practice would be the first. Because if I don’t see the need, or if I don’t see certain types of patients, I don’t bother wasting my time looking into things.”* (P3, GP)

#### Reflective motivation: education delivery to engage health professionals

PHPs discussed aspects of education that increased their motivation to attend and maintained engagement. Professionals frequently received information about CPD programmes from local primary healthcare organizations, and were motivated to attend events from reputable sources without competing commercial interests:

*“Some of the things that I’ve seen sometimes it depends who sponsors them as well. So, I guess, I’m always aware of the WHO Code [International Code of Marketing of Breast-Milk Substitutes], and if there’s any formula companies sponsoring events, I would not attend that and not promote that within the unit.”* (P18, CFHN)

*“The PHN [Primary Health Network] puts up good CPD stuff and it’s much less commercial or not sponsored or anything like that, so it’s not skewed one way or the other, you’re getting an independent voice.”* (P20, GP)

PHPs reported they sought out CPD on topics of personal interest – *“I made a decision probably about two years ago that I was only gonna go to things that I was really interested in*” (P21, GPN) and some particularly valued opportunities to learn from clinicians or consumers with experience dealing with the topic of education:

*“That lived experience from consumers, or from nutritionists and GPs who deal with this all the time. And say, “Oh look, you know, I went in for this and this is what worked for me…” [...] That would be good.”* (P29, CFHN)

While PHPs had reflected on the ease of access to online training options, some preferred face-to-face education, including learning opportunities at conferences:

*“If it’s a workshop or a conference where I feel like there are multiple things that I’d be interested in seeing then I would go along for sure”* (P6, GP)

Regardless of format, several PHPs reflected on the importance of interactivity and preferred interactive education delivery to more didactic methods:

*“What I really like, in terms of professional development, is- I get really tired reading stuff, so if it means I have to sit down and read something and click through something, I probably won’t do it. I much prefer the interactive* – *you’re actually talking with someone or even like a webinar presentation”* (P30, GPN)

## Discussion

These findings highlight that many PHPs, particularly in general practice, did not routinely use guidelines to support their practice related to health promotion and obesity prevention in early childhood. Furthermore, we found almost a quarter of PHPs surveyed had not received any CPD regarding early childhood health behaviours and obesity prevention, with general practice PHPs less likely than CFHNs to have participated in CPD on these topics. Most PHPs were interested in further education to support health promotion and obesity prevention in early childhood, however, self-funding CPD and attending CPD outside of work hours limited opportunities to participate, particularly among GPNs. A considerable lack of education and resources were available to support families from culturally and linguistically diverse backgrounds. Gaps in resources to support bottle-feeding parents and parents with lower health literacy were also highlighted during interviews. Our findings offer insight into PHP preferences for CPD design, including the importance of interactive and flexible modes of delivery, and the need for accompanying parental resources that are visual and available in digital formats.

The current paper is the second to arise from this mixed methods study with Australian PHPs. The first study focussed on examining the current practices, self-efficacy, knowledge and attitudes of PHPs regarding early childhood health promotion and obesity prevention (House *et al.,* 2025). It also examined the barriers and facilitators to embedding health promotion and obesity prevention into routine practice when working with families of young children. The first study identified several barriers to embedding early childhood health promotion and obesity prevention in practice (House *et al.,* 2025). The current paper sought to explore one barrier to implementation in practice in further detail, that is, a perceived lack of knowledge and skills regarding early childhood health behaviours and the need for further training in this area. However, it is important to recognize that several other barriers to embedding early childhood health promotion and obesity prevention in practice exist. These include other health professional level factors (e.g. reluctance to negatively impact the parent-provider relationship by discussing child health behaviours), parent level factors (e.g. competing parental priorities and challenges), and organizational factors (e.g. limited time allocated to preventive care, lack of remuneration for health promotion activities, staffing challenges) (House *et al.,* 2025). Thus, the development of PHP professional development programmes is only one of several important steps in facilitating the implementation of early childhood health promotion and obesity prevention in Australian primary healthcare.

Despite the existence of primary healthcare guidelines relating to promotion of healthy growth and associated health behaviours in children (Dutch *et al.*, 2025), implementation of these guidelines appears to be lacking in Australian primary healthcare. Previous reviews have highlighted that clinical practice guideline development is often not accompanied by sufficient implementation planning, informed by robust theoretical frameworks (Kredo *et al.*, 2016, Liu *et al.*, 2022). To our knowledge, no studies have been conducted in Australian primary healthcare settings to examine the sustainability of clinical practice guideline implementation related to early childhood obesity prevention (Liu *et al.*, 2022, Kredo *et al.*, 2016). A 2016 review examined the potential of digital education delivery to support the implementation of clinical practice guidelines and identified some evidence to suggest that web-based workshops and multi-component interventions can improve knowledge and skills related to the implementation of various clinical practice guidelines (De Angelis *et al.*, 2016). As only about half of PHPs in this survey used guidelines to inform their practice related to growth monitoring, children’s physical activity and sedentary behaviour, the implementation of preventive care guidelines in primary healthcare remains an important area of continued research. In particular, future research should consider PHP perspectives on the current early childhood preventive care guidelines as PHPs’ perspectives regarding the applicability and credibility of guidelines have been identified as important factors that may impact implementation (Wang *et al.*, 2023). Such research may support the development of guidelines and associated implementation tools tailored to the needs of PHPs.

This study highlights a gap in the use of preventive care guidelines, and access to education and resources to support early childhood obesity prevention in the general practice setting. This is significant given that the proportion of young children receiving care in general practice has historically exceeded the proportion receiving care from CFHNs. This disparity in access becomes more pronounced as children reach school age, with most families disengaging from child and family health services but continuing to access general practice (Warren, 2018). PHPs in general practice therefore have ongoing opportunities to educate and support parents with obesity prevention throughout childhood. Workforce challenges, including staff shortages and poor retention, facing the Australian nursing workforce are likely to impact the capacity of child and family health services (Australian Government Department of Health and Aged Care *et al.*, 2024, Victoria State Government and Western Metropolitan Partnership, 2023). In light of this, there may be a shift towards delivering well-child healthcare in general practice. In this context, general practice PHPs must receive adequate training and have access to resources to support child health and development in the early years, as inadequate resourcing in this service setting has implications for child health far beyond obesity prevention.

Health professionals in this study identified a lack of resources and training to support healthy growth and associated behaviours for families from culturally and linguistically diverse backgrounds. This is consistent with earlier Australian research, across health settings, in which health professionals have reported that training to support cultural competence is limited in scope and there is a need for further training to be developed to facilitate culturally responsive care (Komaric *et al.*, 2012, Forbes *et al.*, 2023, Sundararajan *et al.*, 2026). This concerning gap in care is likely to influence satisfaction and engagement with the health system. A previous qualitative study with Arabic- and Mongolian-speaking mothers in Australia described shortfalls in perceived care provided by PHPs in early childhood, including lack of time and clarity about the health system – leading them to prefer to seek advice from doctors in their home country or via the internet (Jawad *et al.*, 2024). Similarly, focus groups with Chinese- and Arabic-speaking mothers in Australia revealed a preference for seeking the support of a bi-cultural family doctor and uncertainty about the child and family health system, which emphasizes the importance of cultural competence across all settings. Parents’ varying level of satisfaction with the health system was reflected in interviews with health professionals who demonstrated differing levels of cultural sensitivity when discussing their practice (Marshall *et al.*, 2021).

Many of the PHPs in this study discussed having access to resources and education to support breastfeeding parents, with three-quarters of CFHNs having received education regarding breastfeeding in the last two years. In comparison, fewer PHPs had received education regarding formula feeding. PHPs described challenges in accessing evidence-based formula-feeding education and resources that were not influenced by infant formula companies. Without sufficient education and resources within primary healthcare, these findings suggest that formula- and mixed-feeding parents may be left seeking support from suboptimal information sources. A qualitative study with Australian parents who formula-fed their infants identified a lack of practical and proactive support from PHPs for formula feeding and feelings of judgement for choosing to formula feed (Appleton *et al.*, 2018). Another quantitative study with parents found that only two-thirds of formula-feeding parents received feeding advice from a CFHN, and only 39% had received any advice from a GPN (Appleton *et al.*, 2020). Such findings suggest that the lack of adequate CPD and resources regarding formula-feeding has negative flow-on effects on formula-feeding parents’ engagement with PHPs.

In the current study, fewer PHPs reported using guidelines, attending CPD and having access to resources regarding physical activity and sedentary behaviour compared to nutritional topics. These findings are consistent with previous research with CFHNs in Australia, which also found that many nurses did not use guidelines or have accesses to resources regarding physical activity and sedentary behaviour (Cheng *et al.*, 2020, Laws *et al.*, 2015). In these studies, they reported a desire for more resources to provide to families on these topics (Cheng *et al.*, 2020, Laws *et al.*, 2015). In one study, nurses also expressed discomfort discussing limiting sedentary behaviour, particularly screen time, due to concern that such advice would be negatively received (Laws *et al.*, 2015). Such findings are also consistent with the international literature, with a systematic review of early childhood obesity prevention practices in primary healthcare settings finding that PHPs less frequently provided support regarding physical activity and reducing sedentary behaviour than nutrition and feeding topics (Ray *et al.*, 2022). This was attributed to limited knowledge of the relevant guidelines as well as a lack of recognition of the importance of physical activity in early childhood (Ray *et al.*, 2022). Our findings, together with the existing body of research, emphasize a need for future research to explore the unique barriers to promotion of physical activity and limited sedentary behaviour in early childhood. This is imperative to guide the development of resources to facilitate holistic promotion of all child health behaviours from early life.

In the current study, none of the themes developed was mapped to automatic motivation. However, preferred topics of CPD that were discussed may provide some insights into emotions that may currently hinder engagement in early childhood health promotion activities. PHPs expressed a desire for CPD to support them to develop the skills to discuss child growth and weight with families and to work sensitively with families of children living with higher weight. This may reflect a level of discomfort in discussing childhood obesity with parents, which echoes reported concerns in other research with Australian PHPs (Canfell *et al.*, 2025). It is important that both knowledge and skills gaps are addressed in CPD developed in this space. This would ensure that early childhood health promotion activities do not perpetuate weight stigma and support PHPs to discuss child growth holistically, avoiding a sole focus on weight and, instead, focussing on promoting overall health (Nutter *et al.*, 2024).

Participating PHPs described a preference for CPD that provided opportunities for interactivity. Such findings are consistent with theories of adult learning, which suggest that adult learning environments should be collaborative and problem-focused, including the use of experiential techniques (Knowles *et al.*, 2015). Similarly, a synthesis of previous reviews regarding features of effective CPD highlighted that interactive modes of CPD are more likely to be effective than more didactic approaches (Samuel *et al.*, 2021). Despite motivation to participate in CPD, barriers to engagement included limited time and funding for CPD and competing organizational priorities, barriers that were also identified in a 2017 survey of US physicians (Cook *et al.*, 2017). Interestingly, in the current study none of the identified barriers to CPD engagement related to social opportunity. This is in contrast to much of the previous literature regarding factors that influence CPD engagement, which has identified that organizational support for CPD engagement is a key facilitator to PHPs completing CPD programmes (Jeong *et al.*, 2018, Walter and Terry, 2021). PHPs identified online learning as offering increased flexibility and accessibility, which is consistent with literature highlighting the potential of online CPD to connect geographically disparate PHPs (Samuel *et al.*, 2021). Today’s landscape is promising for expanding the CPD available to Australian PHPs, as the experience of the COVID-19 pandemic has prompted a shift to online learning platforms. The uptake of these has been rapid among universities but also has the potential to transform the CPD experience (Adedoyin and Soykan, 2023).

## Strengths and limitations

This study adds to a body of literature regarding the implementation of early childhood health promotion and obesity prevention in Australian primary healthcare settings. Earlier studies have primarily focussed on single professional groups (e.g. GPs, CFHNs) and/or have been conducted in small jurisdictions within Australia (e.g. single state) (Cheng *et al.*, 2020, Laws *et al.*, 2015, McMeniman *et al.*, 2011, Robinson *et al.*, 2013). To our knowledge, this is the first national study to examine training needs and preferences for CPD regarding early childhood obesity prevention among a cohort of PHPs working in different disciplines across Australian primary healthcare settings. The mixed methods approach provided opportunities to elicit qualitative explanations for survey findings. The qualitative data collection offered additional flexibility for PHPs to highlight CPD and resource needs that may not have been captured in the survey questions.

The study had several limitations, some of which have been previously reported (House *et al.,* 2025), including potential selection bias, with participants self-selecting to participate in this study potentially representing a group of PHPs with a strong interest in the topics explored. In addition, this study relied entirely on self-report data. This may be affected by attribution bias whereby individuals may be more likely to identify external factors as barriers to their performance of desired behaviours than internal factors (Försterling, 2001). In this study, given the relatively small sample size, particularly the low number of male participants, we were unable to examine the impact of sociodemographic characteristics on PHPs’ training preferences. The high proportion of female participants is not unexpected among CFHNs and GPNs, given that workforce surveys indicate that over 95% of primary care nurses are female (You *et al.*, 2025, Nguyen *et al.*, 2023). However, the perspectives of male GPs are likely under-represented, with almost 50% of GPs being male we would expect a larger proportion of male participants in a representative sample of PHPs (Australian Government Department of Health Disability and Ageing, 2025). In contrast, the age range of included participants appears to be relatively consistent with national workforce estimates, with the majority of participants aged between 40-60 years (You *et al.*, 2025, Nguyen *et al.*, 2023, Australian Government Department of Health Disability and Ageing, 2025). In addition, we did not collect information regarding PHPs’ own weight status and lifestyle behaviours which may influence their responses. As this research was conducted entirely online, perspectives of PHPs with less reliable internet connection, particularly in regional and remote areas, may not have been adequately captured. The survey used in this study has been reviewed for face validity and used in previous research with CFHNs but has not undergone complete psychometric evaluation (Cheng *et al.*, 2020, Laws *et al.*, 2015). The adaptation of the survey for this project was informed by the need to gather PHPs’ perspectives to inform the design of the ‘Connecting the Dots’ early childhood nutrition professional development programme (Karitane, 2025). Therefore, the questions regarding preferences for future CPD programmes focus largely on nutrition and PHPs’ interest in other child health behaviours (physical activity, sedentary behaviour, oral health, sleep) may not have been fully explored.

## Implications for future research, policy and practice

The current findings offer insights into the preferred topics, features and format of CPD to support early childhood health promotion and obesity prevention in Australian primary healthcare settings. However, as noted throughout the discussion, there are several areas that require future research. Further qualitative investigation of PHPs’ views of guidelines regarding early childhood growth and health behaviours may offer insights into the reason for the low use of these guidelines in practice. In addition, our exploration of specific topics for future education was largely focussed on early childhood nutrition. Future research should prioritize more in-depth exploration of training needs regarding other child health behaviours (including physical activity, sedentary behaviour, sleep).

Despite a strong policy imperative for embedding early childhood health promotion and obesity prevention across healthcare settings (Commonwealth of Australia, 2021, Commonwealth of Australia, 2022). The findings of the current study highlight a gap between policy, clinical practice guidelines, and the resources available to support their implementation. Australian PHPs require access to evidence-based education and resources to support early childhood health promotion and obesity prevention. In particular, CPD developers should consider expanding training regarding supporting parents who choose to bottle-feed, providing support to families from culturally and linguistically diverse background, and having non-stigmatizing discussions regarding child growth. However, taken together with our previous research in this cohort (House *et al.,* 2025), it is important that such education and training is accompanied by organizational changes that provide PHPs with increased opportunities to embed early childhood obesity prevention into their routine practice.

## Conclusions

This study offered insights into the training needs of Australian PHPs regarding early childhood health promotion and obesity prevention. The findings indicate that there is a strong interest in further CPD development in this space. In addition to further training, PHPs require access to parent-facing visual resources that are accessible to families with varying literacy levels. The development of future CPD should be informed by the needs of PHPs, considering their busy clinical schedules and preference for self-paced and interactive education modalities.

## Supporting information

House et al. supplementary material House et al. supplementary material

## Data Availability

Data from the current study are not available without additional ethics approval. Research participants were assured that raw data would remain confidential and not be shared.

## Supplementary material

To view supplementary material for this article, please visit https://doi.org/10.1017/S146342362610108X
