# Supplementary material for: Identifying the training needs of Australian primary health professionals to support early childhood obesity prevention: a mixed methods study
Source: Prim Health Care Res Dev. 2026 Mar 27;27:e44. doi: 10.1017/S146342362610108X (PMC13080530; doi:10.1017/S146342362610108X)
Supplement: House et al. supplementary material [file S146342362610108Xsup001.docx]

**Appendix 3 – Supplementary Tables**

**Supplementary Table 1^a^: Demographics characteristics of Australian primary health professionals who participated in an online survey (a) and semi-structured interviews (b)**

1. **Survey participants**

| Characteristic | All participants (n=227) |
| --- | --- |
| Gender | **N (%)** |
| - Male | 9 (4%) |
| - Female | 176 (78%) |
| - Not reported | 42 (19%) |
| Role |  |
| - Child and family health nurse | 104 (46%) |
| - General practitioner | 35 (15%) |
| - General practice nurse | 15 (7%) |
| - Other primary care nurse | 18 (8%) |
| - Other | 15 (7%) |
| - Not reported | 40 (18%) |
| Age |  |
| - 20-29 years | 13 (6%) |
| - 30-39 years | 41 (18%) |
| - 40-49 years | 39 (27%) |
| - 50-59 years | 63 (28%) |
| - 60+ years | 32 (14%) |
| - Not reported | 39 (17%) |
| Years of experience |  |
| - Less than 5 | 54 (24%) |
| - 5-10 | 48 (21%) |
| - 11-15 | 31 (14%) |
| - More than 15 | 55 (24%) |
| - Not reported | 39 (17%) |
| Work hours |  |
| - Full-time | 77 (34%) |
| - Part-time | 110 (49%) |
| - Not reported | 40 (18%) |
| State |  |
| - Australian Capital Territory | 3 (1%) |
| - New South Wales | 110 (49%) |
| - Northern Territory | 18 (8%) |
| - Queensland | 11 (5%) |
| - South Australia | 6 (3%) |
| - Tasmania | 6 (3%) |
| - Victoria | 15 (7%) |
| - Western Australia | 12 (5%) |
| - Not reported | 46 (20%) |
| IRSD Decile |  |
| - 1 | 16 (7%) |
| - 2 | 13 (6%) |
| - 3 | 12 (5%) |
| - 4 | 19 (8%) |
| - 5 | 22 (10%) |
| - 6 | 19 (8%) |
| - 7 | 12 (5%) |
| - 8 | 23 (10%) |
| - 9 | 14 (6%) |
| - 10 | 31 (14%) |
| - Not available | 46 (20%) |
| Socioeconomic Status of work location (based on IRSD) |  |
| - Low | 41 (18%) |
| - Middle | 72 (32%) |
| - High | 68 (30%) |
| - Not available | 46 (20%) |

**b) Interview participants**

| Characteristic | N (total n=28) |
| --- | --- |
| Gender |  |
| - Male | 1 |
| - Female | 27 |
| Age |  |
| - 20-29 years | 1 |
| - 30-39 years | 11 |
| - 40-49 years | 6 |
| - 50-59 years | 6 |
| - 60+ years | 4 |
| Profession |  |
| - Child and family health nurse/nurse educator | 13 |
| - General practice nurse | 5 |
| - General practitioner | 8 |
| - Other | 2 |
| Years of experience |  |
| - Less than 5 | 5 |
| - 5-10 | 10 |
| - 11-15 | 5 |
| - More than 15 | 8 |
| Work hours |  |
| - Full-time | 12 |
| - Part-time | 16 |
| State |  |
| - Australian Capital Territory | 1 |
| - New South Wales | 15 |
| - Northern Territory | 0 |
| - Queensland | 3 |
| - South Australia | 2 |
| - Tasmania | 3 |
| - Victoria | 2 |
| - Western Australia | 2 |
| Socioeconomic Status of work location (based on IRSD) |  |
| - Low | 10 |
| - Middle | 14 |
| - High | 4 |

*Abbreviations:* IRSD – Index of Relative Socio-economic Disadvantage

1. Tables recreated from previous publication using the same data: House, E. T., Kerr, E., Taki, S., Denney-Wilson, E., Baur, L. A., Cheng, H., Rossiter, C., Vlahos, S. & Wen, L. M. 2025. A comparison of early childhood obesity prevention in Australian general practice and child and family health settings: A mixed methods study. Journal of Pediatric Nursing, 81, 97-107.

**Supplementary Table 2: Additional supporting qualitative data**

| Themes | Sub-themes and supporting data |
| --- | --- |
| *Capability (psychological)* | |
| *Equip professionals with the knowledge and skills to confidently promote health* | *“We do a one-day focused workshop on paediatrics and incorporated in that we talk a lot about the first year of life, and some of the issues that can occur within kind of growth and introduction of solids and breast and bottle feeding. Beyond that, there's a little bit of nutritional training, a little bit of food allergy training scattered through. But I think GPs no different to most medical degrees in that you don't really get a lot of nutrition training unless you go looking for it.”* (P26, GP)  *“I think definitely the fussy eating because it is so common and there's so many potential reasons for that as well. So, you know, having a bit of an idea on the kinds of questions to ask to kind of go, alright, this is- sounds like it's more sensory or maybe it's this or maybe it's that. Just to help with what kind of referrals or supports or strategies are gonna be helpful.”* (P28, CFHN)  *Interviewer: “Do staff have access to any training around culturally adapting the nutrition messaging that they're providing to families?”*  *Professional: “No, definitely a gap. And even for our Indigenous families, you know, there's nothing. Yeah, yeah, there's old resources, I know, but there's not current resources.”* (P18, CFHN)  *“As qualified child and family health nurses like, we know about the signs of readiness and things. So, I think it's more about keeping up to date with, you know, any things in like formula changes, you know, there's always new research coming out about like the different types of formulas and things. And all the new fads, you know, like baby-led weaning and all of that”* (P31, Other PHP) |
| *Parent resources to support health promotion discussions* | *“It's really hard to find things on, I guess, overfeeding with formula. So you know, there's like the guidelines on the tin that says, you know, baby should have 5 to 7 bottles a day or whatever, but really there's nothing to say why or anything pictorial, you know, to say don't do this because this can happen, if your baby does need more bottles, you know, maybe there's something else going on. You know, maybe they're asking for something else and not a bottle, you know. So yeah, dealing with those early signs of obesity, I find that there's, like, a lack of resources, definitely.”* (P31, Other PHP)  *“I think very simple daily requirements, in very simple pictorial, you know, fact sheets are very helpful for parents. Colourful, quantities, what they look like, those sorts of things.”* (P15, CFHN)  *“I think those hands-on practical cooking options you know where, you know, easy meal ideas that are nutritious and can be achieved and confidence gained and, you know, the flow on from that I think would be really good.”* (P8, CFHN) |
| *Design resources for real parents* | *“there's a lot of good health podcasts now, and that there's a lot of good people contributing to them and so I'm starting to try and promote that a little bit.”* (P26, GP)  *“Young mums like things at their fingertips, suggesting books doesn't work up, things at their fingertips. […] if we had a, you know, a free app that they could just look at and say this is, you know, this is where we're going. People do- mum’s like directions.”* (P14, GPN)  *“Simplistic information, not too wordy. You know parents don’t need more and more words, they don’t read it, they won’t read it.”* (P2, CFHN)  *“We have for adults, you know, we have like portion sizes and, you know, plates with sort of portions that we’ll set out. I think that they're really useful visual tools for people. I've always found, you know, you can get these little balls that are the size of a baby’s, you know, a newborn baby’s stomach, and then, you know, maybe stomach at 2 weeks and, you know, just to see the tiny size that those are, so I think tools like that are really useful. I haven't seen any of those around for, you know, quite a few years now. We have the, you know, plates that we can give people and say, you know, this is your protein, this is your carbohydrates, this is your, you know, veggies, something like that with child-sized meals on would be good.”* (P21, GPN)  *“We have a few resources that are adapted for our Aboriginal clients, but not really much in terms of culturally and linguistically diverse from overseas and other cultures.”* (P28, CFHN) |
| *Opportunity (physical)* | |
| *Practical CPD solutions for busy clinicians* | *“During the day it's virtually impossible. It would have to be, I suppose, approved by the management that I work for […] it's business-based. So, if it's not going to bring in business or grow the business, it's very difficult to do, unless I took an annual leave day.”* (P27, GPN)  *“I do those [webinars] quite often, but I much prefer if I have the option of watching it later as a recorded webinar, if I have the option of watching it right away. If it's not recorded, probably won't sign up to it.”* (P30, GPN)  *“I think that a lot of it [choice of CPD] would come down to the actual clinician. We do, I personally, try and do quite a bit around breastfeeding, because that's, I think that's a large part of our job and that's something that I enjoy working with mums.”* (P4, CFHN)  *“I think the cost for upskilling with this sort of stuff is a big deterrent.”* (P8, CFHN)  *“I think now because we're all so used to doing Zoom […] we’re very good at logging on and doing those quick, you know, webinar-type education sessions that might go for half an hour […] and I think a lot of people find them helpful 'cause you feel like you're kind of keeping up to date even when you're so busy in your clinical work you can kind of log on and see that.”* (P10, CFHN) |
| *Motivation (reflective)* | |
| *Education delivery to engage health professionals* | *“Hearing from dietitians would definitely be a big bonus for me, because I've got so much to learn from them and I really want to hear what their expertise is that I can then learn from to hand down.”* (P5, CFHN)  *“being regional, anything that we can access here. I mean online is great, but it is really nice to have face-to-face sometimes as well.”* (P28, CFHN)  *“online teleconferences, which if they're interactive is really good. You know, if we have the opportunity to ask questions and have a conversation, that's a lot better. Rather than just like tuning into a conference or anything like that”* (P21, Other PHP) |

*Abbreviations:* CFHN – Child and Family Health Nurse; GP – general practitioner; GPN – general practice nurse; PHP – primary health professionals
